# Supplementary material for: Are carbon emissions trading and green financial instruments synergistic? -Comprehensive quantitative research based on content analysis
Source: PLoS One. 2024 Mar 7;19(3):e0298601. doi: 10.1371/journal.pone.0298601 (PMC10919601; doi:10.1371/journal.pone.0298601)
Supplement: S1 Data — (ZIP) [file pone.0298601.s001.zip › Data/地级市Z_F工作报告.zip!/░▓╗╒/┬φ░░╔╜╩╨/└┤╫╘╣1⁄2╓┌║┼í╛┬φ┐╦╩2╛▌═°í┐_2014.docx]

# **政府工作报告（2014）**

【字体大小：[大](http://zwgk.mas.gov.cn/openness/detail/content/javascript:doZoom(18)) [中](http://zwgk.mas.gov.cn/openness/detail/content/javascript:doZoom(15)) [小](http://zwgk.mas.gov.cn/openness/detail/content/javascript:doZoom(14))】

政府工作报告

——2014年1月6日在马鞍山市第十五届

人民代表大会第三次会议上

市 长   魏 尧

各位代表：

现在，我代表市人民政府，向大会作工作报告，请予审议，并请市政协委员和其他列席人员提出意见。

一、2013年工作回顾

刚刚过去的2013年，是马鞍山发展史上极不平凡、具有特殊意义的一年。这一年，马鞍山长江大桥正式建成通车，开启了一江两岸协调发展的新篇章；这一年，顺利完成全面小康社会计划目标，小康实现程度达99.7%，正朝着建设更高水平小康社会的目标迈进；这一年，多点支撑、多区联动格局逐步完善正在发力，建设“四个中心”、打造“一链一基地四园区”工作取得积极进展，转变经济发展方式迈出重要步伐。一年来，面对复杂多变的宏观形势，在省委、省政府和市委的坚强领导下，我们团结和依靠全市人民，以党的十八大精神为指引，立足过去良好的发展基础，积极应对各种考验，全力推动科学发展，统筹抓好兴实体、稳增长，强平台、促升级，优环境、惠民生等各项工作，较好完成了市十五届人大一次会议确定的主要目标任务，经济社会呈现稳中有进、稳中向好、民生改善、社会和谐的良好发展态势。

预计，全市实现地区生产总值1300亿元，增长11%；财政收入226亿元，增长7.3%；规模以上工业增加值555亿元，增长14.4%；固定资产投资1440亿元，增长20%；社会消费品零售总额300.5亿元，增长14.6%；城镇居民人均可支配收入34500元，增长10.5%；农民人均纯收入13200元，增长14%。被国务院列入全国“老工业基地调整改造城市”、“资源再生型城市”，成功创建首批“国家公共文化服务体系示范区”，全国城市文明程度指数测评居127个参评城市第2名，被评为全国首批“国土资源节约集约模范市”，成为首批“国家信息消费试点市”，连续第八次获得“全国科技进步先进市”称号，入选“全国外贸百强城市”和福布斯“中国大陆最佳商业百强城市”，入围“魅力中国——外籍人才眼中最具吸引力的城市”。

（一）经济发展稳中有进

**“项目提速年”活动扎实开展。**推行重大项目领导领衔、合同管理、公开承诺、会办点评等机制，强化大督查和保平安工作，推进项目提速、工作提效。实施360个重点项目，完成投资760亿元。华菱车架、德国易能环保一期等76个项目建成投产，正崴科技园、信德石头造纸、同心石化等134个项目开工建设，九华路过江隧道、工业机器人等一批重点项目谋划取得积极进展。大力招商引资，围绕“两图一案”，编制主导产业地图、重点企业地图，做细做实招商方案，全年实际利用内资增长25%，外商直接投资增长12%，威博电器、中兴新通讯等60个10亿元以上项目成功签约。要素保障有序有力。完成土地整治32.9万亩，获批建设用地2.8万亩、供地3.2万亩，开工建设标准化厂房150万平方米、基本建成88万平方米。完成征迁拆迁681万平方米，安置房在建5万套、竣工1.7万套。新增各类金融机构8家，金融机构本外币贷款余额1002亿元。直接融资规模163亿元，社会融资总规模520亿元。

**三次产业协调发展。**深入开展服务企业活动，及时出台中小微企业还贷应急周转金、小微企业贷款风险补偿基金等一系列政策措施，支持企业转型升级。工业经济稳步回升，新增规模以上工业企业147户、总数达787户。市与马钢“融为一体”，建立常态化对接机制，马钢加大改革力度，推进主辅分离，实现年度扭亏。山鹰纸业完成重大资产重组。华菱星马、华星化工完成定向增发。建筑业实现产值390亿元，增长25%。积极应对持续高温干旱天气，农业平稳发展，规模经营水平大幅提高，新增各类新型经营主体368个，土地流转比重提高9个百分点、达46%，10个现代农业示范区完成投资18亿元。服务业健康发展，完成增加值370亿元。新建4条特色商业街，5座城市综合体主体完工，“淘马鞍山”等3个电子商务平台建成上线，“新网工程”建设深入推进。成功举办第二届海峡两岸（马鞍山）电子信息博览会等各类展会。完成旅游项目投资102亿元，旅游业总收入达122亿元。完成房地产开发投资265亿元、增长25%，竣工商品房242万平方米、增长18%，销售285万平方米、增长26%。

（二）多点支撑成效明显

**县区园区实力增强。**县区生产总值平均增长12%，占全市比重达70%。含山县财政收入等主要经济指标三年实现倍增，被列入全省深化农村综合改革示范试点县。和县获批全省首批农村商品流通服务体系建设试点县，成为全省泵阀专业商标品牌基地。当涂县跻身全国中小城市百强县，县经开区入选省创新型园区十强。花山区濮塘休闲度假区总体规划获批，软件园入驻企业超百家。雨山区规模以上工业增加值增幅达35%，磁性材料产业集聚区初具规模。博望区新城区规划和基础设施建设加快推进，获准筹建全国剪折机床产业知名品牌创建示范区。完成市经开区、慈湖高新区、示范园区管理体制改革，推行扁平化管理、公司化运作，发展活力进一步增强。市经开区开工建设亿元以上项目28个，马钢晋西轮轴一期等18个亿元以上项目竣工投产。慈湖高新区完成工业性投资129亿元，中小企业科技孵化器投入运营。郑蒲港新区30平方公里起步区框架拉开，北京御香苑等一批项目签约。示范园区绿色建筑装饰材料产业园实现当年开工、当年运营。秀山新区城市形态初显，秀山湖公园对外开放。滨江新区重点文化旅游项目积极推进。6个园区、新区完成固定资产投资突破500亿元，占全市34.8%，增长23%。

**转型升级步伐加快。**战略性新兴产业实现产值390亿元，增长33%，机器人产业区域集聚发展试点获国家批准。新认定高新技术企业44户、国家火炬重点高新技术企业3户、高新技术产品235个、国家和省重点新产品10个。深化产学研合作，中钢天源院士工作站成立，安工大工业技术研究院揭牌，新增6家企业博士后工作站。授权发明专利258件，华骐环保获省发明专利金奖。认定省著名商标48件，和县辣椒、当涂园艺鲜桃入选中国地理标志证明商标。华菱星马获得国家出口免验企业资格、省政府质量奖和省科技进步一等奖。华星化工入选首批“国家级知识产权示范企业”。新增省级服务业集聚区2家，总数达6家、居全省第一。民营经济蓬勃发展，新发展个体工商户1.3万户、私营企业3085户。对外合作得到加强，实现外贸进出口37亿美元，完成外经营业额2.5亿美元，口岸核心能力建设通过国家验收。博望区获批省级出口机床暨刃模具质量安全示范区，和县获批省级出口蔬菜质量安全示范区。参加南京都市圈城市发展联盟，马巢、马溧高速建成通车，马滁高速加快建设，示范园区江东大道对接芜湖徽州大道、当涂赤铸山大桥等项目进展顺利，宿马产业园完成固定资产投资30亿元，区域一体化合作积极推进。

（三）城乡环境不断优化

**基础设施加快建设。**完成投资264亿元，增长19%。宁安城际铁路马鞍山段加快建设，合福铁路含山段进展顺利，城市轨道交通线网规划编制完成。205国道南段改建基本建成、北段改造加快推进，314省道改造全面完工，206省道、226省道改造稳步推进。郑蒲港一期2个万吨级码头主体完工，港口集团太平府综合码头建成。新建城区主干道路17公里，升级改造县乡公路62公里，完成农村危桥改造20座，在全省率先实现农村清洁工程全覆盖，改造农村危房8600户。完成滁河防洪治理年度计划和38公里中小河流治理工程。建成220千伏霍里输变电等7项电力重点工程，供电保障能力提升。新增天然气用户2.5万户、自来水用户2.8万户。新开通城市公交线路2条，新投入纯电动公交车30辆。

**城乡面貌明显改观。**市体育会展中心加快建设，部分场馆投入使用。科技馆、青少年宫、妇女儿童活动中心“新三馆”开馆。慈湖河中游综合整治工程顺利推进，城市出入口环境整治成效明显，南湖公园地下停车场工程主体完工，花果山公园基本建成，新增城市绿道27公里，建成5座街头游园。完成14个老旧小区整治。完成城区主要道路门头招牌改造，数字化城管系统投入运行。美好乡村建设深入推进，建成26个省级重点示范村、50个市级示范村。推进森林增长工程，新增人工造林15万亩、森林长廊96公里。抓好节能减排，实施减排重点工程53项，建成污水管网19公里，新建改建脱硫脱硝设施14个，启动生活垃圾焚烧发电项目，万元GDP能耗下降5.5%，主要污染物减排总量控制在省下达指标以内。

（四）公共服务水平提升

**民生工程扎实推进。**投入资金36.5亿元，增长21%。大力促进就业，鼓励扶持创业，新增城镇就业5万人，期末城镇登记失业率2.8%，兑现稳岗补贴1.7亿元。县区基本医疗、失业、工伤、生育保险实现市级统筹，五项社保新增参保2.4万人。被征地农民养老保障金每月增加60元。提高城乡低保、农村五保标准，增幅均超过10%。完成养老项目21个，新增社会养老床位2370张，城镇社区助老服务站实现全覆盖。保障性住房新开工2万套、竣工1.2万套，各项考核指标均位居全省前列。住房公积金归集和使用金额再创历史新高。物价水平总体稳定。

**社会事业协调发展。**学前教育三年行动计划全面完成，启动实施教育信息化三年行动计划，博望区试点开展中小学校长职级制改革，二十二中、当涂一中、培正学校新校区投入使用，在全省率先实现中职教育学费全免。完成市博物馆展陈提升改造，建成10个自助图书馆，完成5个省级农民文化乐园试点村建设。节俭办好李白诗歌节，举办全国诗歌大奖赛，入选国家高水平体育后备人才基地。凌家滩遗址入围国家考古遗址公园立项名单，太白楼入选全国重点文物保护单位。市、县级公立医院改革深入推进，基层卫生服务体系建设得到加强，秀山医院加快建设，三县新建的人民医院投入使用。企业一套表改革工作扎实推进，做好第三次全国经济普查准备工作。粮食收购创历史新高，粮食储备管理工作不断加强。广播电视、新闻出版、文学艺术、哲学社会科学进一步繁荣。档案、地方志工作水平不断提高。外事、侨务、港澳台，气象、地震、防灾减灾工作得到加强。青少年、妇女儿童、老龄工作全面进步。红十字志愿服务工作创新发展，残疾人保障工作取得新进步。

**社会治理工作不断加强。**扎实开展全国和谐社区建设示范城市创建工作，深入推进社区网格化管理，全市“精品”、“四优”社区达到35个。坚持领导干部开门接访、重点约访、带案下访，健全三级信访工作平台和联合接访机制，全力化解信访积案，信访秩序不断规范。推进“平安马鞍山”建设，严厉打击各类违法犯罪，不断完善社会治安防控体系，全年刑事案件同比下降2%，成功侦破“4.14”特大跨国电信诈骗案，群众安全感进一步提升。积极稳妥推进户籍制度改革，进一步放宽城镇户口落户政策。社区矫正中心全面建成并有效运行，县区安置帮教基地实现全覆盖。加大执法监督力度，安全生产形势总体稳定。人口和计划生育工作全面加强，综合考评居全省前列。深入开展食品药品安全专项整治，食品药品市场安全有序。完善应急管理体制机制，预防和处置突发事件能力明显增强。民族团结、宗教和睦局面进一步巩固。国防教育、国防动员、人民武装、人民防空、双拥优抚工作得到加强。驻马部队和广大民兵预备役人员在促进社会稳定、支持地方建设等方面作出了重要贡献。

（五）政府建设得到加强

坚持依法行政，认真执行市人大及其常委会决议、决定，自觉接受市人大法律监督、工作监督和市政协民主监督，认真办理人大议案、建议、意见和政协提案，注意倾听和吸纳各民主党派、工商联、无党派和社会各界人士的意见建议。密切与工会、共青团、妇联等人民团体的联系。完善政府工作规则和决策咨询机制。完成公路管理体制改革，整合地方海事和港口管理机构，深化事业单位人事分配制度改革，继续完善投融资、工程建设、招标采购等六项管理体制改革，成立市公共资源交易中心。推进行政审批制度改革，审批事项精简53.4%，保留事项办理时限平均压缩59.4%。整合44条服务热线，实现“12345一个号码找政府”。政府信息公开工作全面加强。改进目标考核机制，有效发挥激励作用。强化问题意识，在近年来解决一大批涉及土地、征迁、用电等重难点问题基础上，又梳理解决了17项疑难问题。强化重点领域、关键环节的行政监察和审计监督，全面推进廉政风险防控，严控“三公”经费支出，树立为民务实清廉的良好形象。

回顾总结去年工作，需要特别报告的是，地区生产总值增长11%，未能实现12%的目标；社会消费品零售总额增长14.6%，未能实现15%的目标；城镇居民人均可支配收入增长10.5%，未能实现13%的目标。究其原因，主观上是我们的工作还有不到位的地方；客观上是由于宏观形势复杂多变、经济下行压力加大，我们自身仍处在艰难转型、负重前行的关键时期。还有，城镇居民人均可支配收入由于基数较高，继续保持快速增长难度加大。

各位代表，过去一年的成绩来之不易。这是在省委、省政府和市委的坚强领导下，全市人民奋发进取、团结拼搏的结果。在此，我代表市人民政府，向辛勤工作在全市各条战线上的广大干部群众，向市人大代表、市政协委员、离退休老同志，向市各民主党派、工商联、无党派人士、人民团体和各界人士，向中央和省驻马单位、人民解放军、武警官兵和人民警察，向所有关心、支持、参与马鞍山建设发展的境内外投资者、海内外朋友们，表示衷心的感谢和崇高的敬意！

在总结成绩的同时，我们也清醒地认识到，发展中还存在一些问题，主要是：经济总量不够大，尤其是工业经济增长不快，规模以上企业数量偏少，面对日益激烈的区域竞争，要实现本届政府提出的“总量争三强”目标任重道远；产业结构不优、集中集聚集约程度不高，推动经济转型升级的大项目、好项目还不够多，招商引资力度还需加大；此外，土地、资金、环境等要素制约依然存在，城乡区域发展不够平衡，影响社会和谐稳定的矛盾仍然较多，政府的创新能力、执行能力、法治思维、服务意识还需要进一步增强，投资环境还需要进一步改善。对这些问题，我们将高度重视、认真解决，不辜负各位代表和全市人民的期望！

二、2014年工作总体安排

今年是全面贯彻落实党的十八届三中全会精神、全面深化改革的第一年，是实施“十二五”规划的关键之年，做好今年工作意义重大。我们要看到，世界经济在较长时期内可能处于低速增长状态，我国经济也由高速增长转向中高速增长阶段，经济运行中不稳定不确定因素依然存在。我们更要看到，中央提出继续坚持稳中求进工作总基调，继续实施积极的财政政策和稳健的货币政策；省委、省政府也明确指出我们仍处于大有作为的黄金发展期、转型升级的战略机遇期、深化改革的重要窗口期，宏观环境总体有利。同时也要看到，近年来，我市抢抓重大发展机遇，优化城市总体布局，始终坚持转型发展、加快发展、和谐发展，各项工作全面推进，为经济持续健康发展打下了坚实基础，提供了新的动力。我们要准确把握宏观形势，深刻认识市情特征，因势利导，主动作为，突出重点，统筹兼顾，牢牢把握新一轮转型升级、加快发展的主动权。

今年政府工作的总体要求是：全面贯彻落实党的十八大、十八届三中全会和中央、全省经济工作会议精神，按照打造“三个强省”、建设美好安徽的统一部署和要求，围绕转型升级、加快发展，坚持稳中求进、改革创新，深入开展“项目突破年”活动，扎实推进“一链一基地四园区”建设，着力做大经济体量、提高经济发展的质量和效益，着力建设生态马鞍山、打造宜业宜居生态环境，着力推进文化强市建设、彰显城市文化魅力和活力，努力保障和改善民生，努力加强和改进政府自身建设，促进经济持续健康发展、社会和谐稳定。

综合考虑，今年经济社会发展的主要预期目标为：地区生产总值增长10%，财政收入与地区生产总值同步增长，固定资产投资增长18%，社会消费品零售总额增长13%，城镇居民人均可支配收入增长9%，农民人均纯收入增长12%，城镇登记失业率控制在4%以内，人口自然增长率、单位生产总值能耗、主要污染物排放总量控制在省下达指标以内。

完成以上目标任务，必须按照市委八届十一次全会总体部署，始终坚持转型升级不动摇，做到投入讲产出、建设重管理、增长有效益，加快转变经济发展方式，促进经济提质增效升级；始终坚持加快发展不动摇，把发展作为解决所有问题的关键，保持经济增速在合理区间平稳较快运行，推进各项工作持续走在全省前列；始终坚持聚焦工业不动摇，围绕做大体量、提升质量，突出服务企业、招商引资，强化科技、金融、人才支撑作用，立足市情走好新型工业化发展道路；始终坚持改革创新不动摇，把改革创新贯穿于经济社会发展的各个领域各个环节，着力在重要领域和关键环节取得实质进展，以改革促进创新发展，以创新驱动经济升级；始终坚持民生优先不动摇，把群众对美好生活的向往，作为政府工作的努力方向，多谋民生之利，多解民生之忧，促进社会公平正义，确保社会和谐稳定，努力让全市人民过上更好生活。

三、着力做大经济体量

经济建设是政府的中心工作，着力做大经济体量，切实提高经济发展的质量和效益，是我们重中之重的任务。

积极扩大有效投入。把加大项目投资力度作为做大经济体量的主抓手，力争在项目数量、体量、结构、签约落地、建设进度五个方面实现突破。实施616个重点项目，完成投资850亿元，增长11.8%。加快推进康佳电子、一重机床、日发纺机等244个续建项目，开工建设中国电子科技产业园、飞毛腿电源等186个项目，建成投产马钢1580热轧、西安开米一期等115个项目，认真谋划山鹰200万吨造纸、宁申石化等186个前期项目。大力招商引资，着力做大增量，力争全年实际利用内资和外商直接投资均增长10%以上，引进10亿元以上重大项目不少于20个。加大技改投入，做强做优存量，实施利洁时医药产业园、中核钛白等149个亿元以上技改项目，完成投资237亿元。鼓励和促进民间投资发展，释放社会资本巨大潜力。加强政府性投资管理，坚持量力而行、尽力而为，分清轻重缓急，统筹项目开工和建设的时序进度。强化要素保障。全面完成工矿废弃地复垦利用试点任务，完成土地整治20万亩，获批建设用地2.5万亩。规范征迁拆迁工作，统筹进度和效益，完成拆迁面积300万平方米。根据可持续发展要求，科学合理确定政府融资规模和结构，创新融资工具，强化融资管理，严格举债程序，防范债务风险。

全力聚焦工业经济。把推动工业经济发展作为做大经济体量的突破口。出台工业经济倍增行动计划及其配套政策。全年新增规模以上工业企业180家，实现规模以上工业增加值600亿元，实施亿元以上重点工业项目149个、其中5亿元以上项目34个，完成工业性投资780亿元、增长20%。加快园区转型升级，突出主导产业，明晰功能定位，创新体制机制，强化目标管理，实行绩效考核，加快标准化厂房建设，搭建各类公共服务平台，打造低成本的投资环境。支持市经开区申报国家新型工业化产业示范基地、慈湖高新区创建国家循环化改造示范试点园区、当涂经开区创建国家级开发区、雨山经开区申报国家级再制造产业示范基地。做大做强铁基新材料高新技术产业链，推进机器人产业区域集聚发展试点，重点扶持重型装备制造、汽车及零部件、电子信息、节能环保及绿装建材等主导产业发展，力争战略性新兴产业产值增长20%以上。全面深入持久开展服务企业活动，实行园区为入园企业全程代理行政审批服务。深化市与马钢常态化对接和联合招商机制，支持马钢继续推进主辅分离、整合矿产资源、深化内部改革和管理。支持华菱星马打造汽车核心零部件产业基地，推进山鹰纸业进一步扩能达效，扶持AVK公司打造亚洲最大阀门生产基地，推动以科达洁能清洁煤气化技术为基础的绿色建材产业园发展。建设中小企业服务平台，抓好“个转企”和“规下升规上”工作。运用信息化途径和手段，把推动信息化与工业化深度融合落实到具体产业、具体企业和具体产品上。完善“质量强市”建设体系，支持博望区建设国家级机床产品检测检验中心。

提升创新驱动能力。把实施创新驱动战略作为做大经济体量的动力源。抢抓安徽成为全国创新型试点省的重大机遇，围绕推进科技成果资本化、产业化，更加突出企业创新主体地位，注重加强与大院名所合作，积极引进各类投资机构，加速集聚创新创业人才，促进经济与科技、金融、产业、人才紧密融合，在全社会营造鼓励创新、支持创新、包容创新的浓厚氛围，打造区域创新高地。积极培育和大力引进产业研究院和研发机构，建立产学研协同创新机制，重视工业设计在产品开发中的作用，实施科技创新券制度，加速推进创新要素向企业集聚。支持华菱星马申报国家工程技术研究中心。授权发明专利280件，促进专利技术产业化。加强知识产权运用和保护，确保国家知识产权示范市培育期创建通过验收。大力推进商标战略，帮助企业培育自主品牌，提高企业市场竞争力。发挥政府引导资金的杠杆作用，吸引实力较强、运作规范的各类投资基金到我市发展，推动更多科技成果在我市转化。积极开展专利、商标权质押等融资创新，引导金融机构加大对科技型中小微企业信贷投放力度，确保金融机构本外币贷款余额突破1100亿元。支持企业上市融资，推动更多科技型中小企业进入“新三板”和省区域股权交易市场。加大人才引进力度，实施技能振兴专项行动，加快领军型、创新型人才和高层次、高技能人才的引进和培养，鼓励和吸引高端人才来我市创新创业，力争通过引进一个团队，催生一个产业，带动一批企业。

加快发展现代农业。以促进农民增收为目的，健全完善现代农业产业体系。培育壮大优质粮油、生态水产、特色畜禽、精品果木、精细蔬菜等优势主导产业，大力推进现代农业示范区和农产品加工区建设，努力打造绿色食品供应中心，农业产值增长10%，规模以上农产品加工产值增长18%。以推进“一村一品”为抓手，加快构建新型农业经营体系。积极培育家庭农场、专业大户、专业合作社和农业企业，壮大新型经营主体，鼓励发展多种形式规模经营，探索开展土地信托和预流转，新增土地流转面积10万亩以上。以加强农业科技服务为重点，着力加强农业社会化服务体系建设。完善农业基础设施，提升农业机械化水平。加强与科研院校合作，推进良种良法的运用。转变农业发展方式，大力发展节水农业、循环农业和生态友好型农业，促进农业可持续发展。加快粮食仓储项目建设，推进主食产业化。加强农资市场监管，保障农产品质量安全。以深化农业农村改革为动力，全面激发农业农村发展活力。扎实推进含山县农村综合改革省级示范试点工作，加快农村各类产权确权登记和集体资产股份制改革，全面推进农村信用体系建设，建立覆盖全市的农村综合产权交易平台，打通农村资产的市场价值实现渠道。

大力发展现代服务业。推进制造业与服务业融合发展，鼓励引导制造业企业抓住“营改增”扩围的机遇，推进主辅分离，提高生产性服务业专业化、社会化水平。积极推进“一基地四园区”建设，筹备建设江海联运枢纽中心大厦，开工建设钢铁生产交易中心及配套仓储加工物流产业园、汽车文化产业园，加快建设示范园区绿装园检测中心，建成软件园一期，启动软件园二期，努力打造安徽软件名城。大力促进信息消费，支持发展电子商务、云计算等新兴业态，积极引进领军企业，培育“淘马鞍山”发展壮大，加快建设花山区电子商务产业园。支持社会力量承办展会，促进会展经济发展。加快秀山新区金融街建设，引进20家金融服务、中介机构入驻。筹备设立首家民营银行——江东银行，完善县区国有担保体系，规范发展小额贷款公司，促进保险行业健康发展，支持发展互联网金融、金融租赁、消费金融等新型金融业态。大力发展现代物流业，以列入国家物流园区发展规划二级节点城市为契机，积极争创省级示范物流园区。加快主城区商圈建设，建成万达广场等4座城市综合体，推进三国文化街等4条特色商业街建设。推进“新网工程”建设，提升农村服务网络建设水平。认真落实权利平等、机会平等、规则平等的要求，为民营经济发展营造更优更好环境，激发全民创业和大力发展民营经济的新热潮。积极发展假日经济，着力培育城市夜间消费市场，逐步提升休闲旅游、文化娱乐、体育健身对消费的拉动作用。推动建筑业提质增效，支持十七冶集团申报特级资质。完善社会养老服务体系，加快发展健康养老服务产业，争取列入养老服务业发展试点城市。

提升对外开放水平。抓住国家扩大内陆开放的有利时机，充分利用两种资源、两个市场，着力提高经济外向度。深入研究上海自贸区体制、规则、政策以及对我市的影响，积极跟进、主动对接、为我所用。认真谋划选定一些领域实施“负面清单”制度，让各类市场主体充分竞争和发展。围绕“两图一案”，创新招商方法，突出工业项目，主攻重点区域，完善考核机制，更加注重推进央企省企、知名民企、世界500强和对台招商活动，更加注重坚持引资和引技、引智相结合，以招商引资提质增效推动全市经济转型升级。转变外贸发展方式，提高贸易便利化水平，培育更多外贸市场主体，完成进出口总额37亿美元。扩大先进技术、关键设备和重要资源进口，支持马钢、十七冶、建诚国际等龙头企业走出去，建立生产基地和营销网络，承揽对外承包工程。积极推动综合保税区申报工作，推进长三角区域大通关建设，争取自贸区建设试点。拓展区域经济交流合作，抢抓长江经济带建设机遇，主动融入长三角，参与城市群分工，扩大宁马、合马、马芜、马宿合作成果。

四、着力建设生态马鞍山

牢固树立尊重自然、顺应自然、保护自然的生态文明理念，坚持“组团式、网格化、田园城市”发展战略，着力建设精致秀丽、宜业宜居的生态马鞍山。

打造宜居城市。坚持规划引领，按照生产空间集约高效、生活空间宜居适度、生态空间山清水秀的要求，启动城市总体规划修改，完善各类专项规划修编，提升城市景观设计水平，自觉维护规划权威。科学有序推进城镇化健康发展，合理设定城镇开发边界，坚持走内涵发展和品质提升之路。加大环山、环湖退让力度，严格控制超高建筑，推进秀山花博园建设，新建改造12个街头游园，新增城市绿道95公里，让城市融入大自然，着力塑造山水园林城市风貌。注重在城市规划建设中保护历史文化遗产，延续城市历史文脉。重视各类地下管网建设和改造，实施黄家塘等5条水系和9处易涝点整治，提升城市防涝能力。加快保障性安居工程建设，新建2.4万套、竣工8000套，推进廉租房、公租房并轨运行。加大安置房建设力度，新建续建5.3万套、竣工2.6万套。稳步推进城中村改造，努力改善群众居住条件。把郑蒲港新区作为跨江发展突破口，加快基础设施建设，打造“产城港一体”的现代临港新城。加快博望区建设，完善城市功能，促进产业集中，着力集聚人气。支持三县县城和重点镇建设，培育发展各具特色的产业体系，营造良好就业和生活环境，推进以人为核心的新型城镇化。依托数字化城管系统，提高城市管理信息化、精细化、科学化水平。

提升通行能力。认真谋划新的过江通道，重点推进九华路过江隧道项目前期工作，力争取得积极进展。推进宁安城际铁路、合福铁路建设，争取北沿江铁路省、市共建，做好城市轨道交通与南京对接工作。做好港口岸线资源规划、开发和保护，建成郑蒲港一期，推进二期项目申报，加强与沿江重点港口合作，用好长江黄金水道资源，打造江海联运枢纽中心。全面完成205国道南段改建工程，加快205国道北段和206、226省道改造进度，推进马滁高速建设，开工建设367省道，做好313省道、慈湖河路升级改造项目前期工作。加快江东大道南延、霍里山大道南延工程建设，续建九华东路、丰收路，打通主城区与各组团之间快速通道。改善城市交通出行条件，大力发展公共交通，缓解局部节点交通拥堵状况。

建设美好乡村。把美好乡村建设作为惠及全市农民的最大民生工程和统筹城乡发展的载体工程。完善村庄布点规划，以改善农村人居环境为着力点，实施铁路、公路、江河沿线以及城市、省际、景区周边等“三线三边”环境整治，扎实推进7条示范线、5个示范区、22个中心村、33个示范村建设。坚持群众主体、因地制宜、注重实效，注意保留村庄原始风貌，慎砍树、不填湖、少拆房，重点开展垃圾、污水治理和危房改造，对规划保留的村庄尽可能在原有基础上改善居民生活条件。改造5000户农村危房，建成43座农村污水处理设施，改建50公里农村公路，改造20座农村危桥，完成25公里中小河流治理工程，推进农村小型水利工程改造提升。注重把产业发展、农民增收与村庄整治同步规划、统筹推进，加强公共服务设施建设，努力实现村美人富、充满活力的目标。

保护生态环境。以创建生态市为抓手，修改完善生态市建设总体规划，认真起草创建工作行动计划，切实抓好重点领域环境整治工作，努力营造人人关心生态、人人参与环保的浓厚氛围，共同打造山清水秀、天蓝地绿的美丽家园。节约集约利用资源，降低能源消费总量，积极淘汰落后产能，严控新上高耗能、高排放项目。支持市经开区创建国家级生态工业园。强力推进大气污染防治。抓好钢铁、电力、水泥等重点行业脱硫、脱硝、除尘设施建设，提高现有减排设施运行效率，全面推行清洁生产；综合治理城市扬尘，抓好渣土车整治工作；推进千万亩森林增长工程，完成造林绿化面积16万亩。加快推进慈湖河中游综合整治工程，开工建设水阳江下游防洪治理、双摆渡泵站改扩建、裕溪河治理等重大水利工程，积极推进长江干流马鞍山河段治理二期、石臼湖水利枢纽等重大水利项目前期工作。加快污水处理厂外部管网配套及泵站建设，提高收水能力和运行效率。改进全社会用水方式，建设节水型社会。加快生活垃圾焚烧发电项目建设。深入开展国土“四模创建”活动，加强矿产资源管理，强化基本农田保护，推进土地节约集约利用。

五、着力推进文化强市建设

文化是一座城市的灵魂，也是区域发展的重要竞争力，更是人民群众的精神家园。要推动文化产业快速发展与文化事业全面繁荣，积极构建现代公共文化服务体系，不断满足广大群众日益增长的精神文化需求。

大力发展文化产业。出台文化产业发展行动计划，通过政策扶持、项目带动、企业培育，努力把文化产业培育成为全市经济的支柱产业，使文化消费成为拉动经济发展的重要力量。制定文化产业扶持政策，提升全市文化产业规模化、集约化、专业化水平，力争文化产业增加值突破50亿元。深化文化体制改革创新，建立健全现代文化市场体系，组建市文化资产管理公司，积极培育和引进各类文化企业。实施重大项目带动战略，积极推进濮塘文化产业园、台湾正崴文创中心、烟墩山文化创意产业园等项目前期工作，加快采石镇改造、郑蒲港白桥老街改造、凌家滩国家考古遗址公园、西楚霸王文化产业基地、明清文化园、东湖艺谷创意园等重点项目建设。加强文化人才队伍建设，重视培养和引进高端文化人才和文化专业人才。促进文化与科技融合，培育发展传媒影视、娱乐演艺、动漫游戏、数字出版、工艺美术等新型文化业态。

全面繁荣文化事业。大力加强社会主义核心价值体系建设，深入推进公民道德建设，切实抓好未成年人思想道德建设，全力做好第四批全国文明城市复评迎检工作。自觉弘扬工业文化，传承工业文明，在全社会营造重视工业、鼓励创业、崇尚创新的浓厚氛围。巩固国家公共文化服务体系示范区创建成果，推进市体育会展中心等公共文化设施建设，新建一批城乡阅报栏，扩大公共文化场馆免费开放范围，提升管理水平，推动公共文化资源共建共享。实施文化惠民乐民工程，继续办好李白诗歌节、“江南之花”等群众文化活动，支持社会团体自办文化活动，提升《千羽锦》等原创舞台剧水平，创作《追梦五千年》等文艺作品。将公共文化服务向农村延伸，推进农民文化乐园建设，整合文化活动室、农家书屋等设施，建设农村综合性文化服务中心。完善群众评价和反馈机制，推动文化惠民项目与群众需求有效对接。加强文化遗产保护，开展可移动文物普查工作。

推动文化旅游融合发展。抢抓皖南国际文化旅游示范区建设机遇，整合一江两岸文化旅游资源，着力打造面向长三角地区的运动健康休闲旅游中心。坚持项目带动，通过政府推动、市场运作，引进国内外知名大企业、大公司参与我市旅游资源开发和项目建设，重点抓好太白生态旅游岛、濮塘海信体育休闲产业园、郑蒲港国色天香游乐园、含山昭关温泉旅游度假区、和县天沐香泉养生谷、当涂大青山李白文化旅游区、博望横山生态旅游区等重点旅游项目建设。扶持影星银幕、古床博物馆、洪滨丝画等地方特色文化旅游企业做大做强，鼓励民间资本开发各具特色的文化旅游产品。探索旅游管理体制改革，激发旅游业发展活力。推进景区提档升级，发展乡村旅游经济，办好“课本上的马鞍山”等旅游主题活动，提升旅游配套服务水平。加强区域融合、要素融合、业态融合，丰富旅游产品结构，延伸旅游产业链，提升旅游竞争力。

六、努力保障和改善民生

把保障和改善民生放在首要位置，多做雪中送炭的重点民生工作，更加注重发挥市场和社会作用，满足群众多样化需求，不断增强人民群众的幸福感和满意度。

努力办好每件民生实事。组织实施好省里统一部署的33项民生工程，在此基础上，市政府将重点做好8件民生实事，包括：城市公交服务升级、提供城市公共自行车服务、实施部分菜市场标准化改造、建设废旧物品回收分拣中心、改造提升部分老旧小区、加强高污染车辆限行管理、完善急救一体化调度指挥平台、推进教育信息化工程。通过办好每一件顺民心、合民意，群众看得见、摸得着的具体实事，让群众更多享受到改革发展的成果。完善就业创业服务体系，建成启用“360行，行行出状元”综合信息化服务平台。着力做好高校毕业生、农村转移劳动力、城镇就业困难人员、退役军人等重点人群就业服务，提高就业质量，实现城镇新增就业4.2万人。按照保基本、兜底线、促公平、可持续的要求，进一步完善社会保障体系。实施县域城镇居民医保与新农合并轨，加快推进社会保险城乡人群全覆盖。加强社会保险基金征缴和监管，确保社会保险待遇按时足额支付。创新劳动关系协调机制，规范收入分配，切实维护劳动者权益。动员组织社会力量参与社会救助，提升社会福利中心功能，不断加强红十字、慈善、残疾人保障等工作，全面提高社会救助水平。完善住房公积金制度。抓好“菜篮子”工程。保持价格总水平基本稳定。

推进社会事业改革发展。深化教育综合改革，促进教育公平发展、均衡发展、特色发展，努力办好人民满意教育。启动第二个学前教育三年行动计划，积极推进中小学及幼儿园集团化办学，扎实推进农村教学点标准化建设，支持郑蒲港新区、博望区各建一所普通高中。加快现代职业教育体系建设，深化产教融合、校企合作，科学设置专业，提高培训实效，努力培养更多适应我市主导产业发展需要的高素质劳动者和技能型人才。巩固扩大基层医改成果，重点落实村医保障工作，提高县级公立医院服务能力。加快秀山医院建设，开工建设市妇幼保健院门诊楼，积极谋划郑蒲港医院，支持非公医疗机构发展。创新市级公立医院集团化管理模式，推进与基层医疗机构分工协作机制，基本建成市级区域卫生信息平台。稳定低生育水平，提高出生人口素质，促进人口长期均衡发展。深入推进全民健身实施计划，积极备战省第十三届运动会。做好第三次全国经济普查工作，夯实基层统计基础。实施城乡气象防灾减灾保障工程。推进外事、侨务和对台工作健康发展，做好档案、地方志等工作，支持工会、共青团、妇联开展各项工作。

促进社会和谐有序充满活力。全力争创全国和谐社区建设示范城市，扎实推进社区管理体制改革，加强村居干部职业化建设，推进公共服务、市场服务、社会服务资源和力量下社区、进网格。推动社会组织健康有序发展，努力把更多社会组织培育成为合格的公共服务提供者。畅通和拓宽群众诉求表达渠道，借鉴“枫桥经验”，夯实信访基层平台，健全及时就地解决群众合理诉求机制。创新立体化社会治安防控体系，提高网络安全管理水平，依法严密防范和惩治各类违法犯罪活动。推进社区矫正、安置帮教和法律援助工作，完善人民调解、行政调解、司法调解联动的工作体系。加强安全生产监管，建立隐患排查治理体系和安全预防控制体系，改进明查暗访方式，切实防范各类安全事故发生。深化完善工商、质监、食品药品监管体制改革，加强监管能力建设，着力营造安全、放心的消费环境。完善应急指挥平台和预警监测体系，加强应急避难场所和救援队伍建设，全面提升预防和处置突发事件能力。推动民族团结进步事业健康发展，维护宗教领域和谐稳定。加强国防教育、国防动员、国防后备力量、人民防空建设，推动军民深度融合发展。

七、努力加强和改进政府自身建设

全面完成今年目标任务，向全市人民交上一份满意答卷，需要政府全体工作人员牢记责任、提升能力，夙夜在公、勤勉工作。

推进法治政府建设。坚持依法治理，运用法治思维和法治方式，让权力在法治轨道上运行，营造公平公正的发展环境。自觉接受市人大法律监督、工作监督和市政协民主监督，认真听取各民主党派、工商联、无党派人士和人民团体意见建议，主动接受社会公众和新闻舆论监督。围绕全面正确履行政府职能，开展新一轮政府职能转变和机构改革，加快事业单位分类改革，加大政府购买服务力度。明晰政府权力清单，承接落实好上级取消、下放的审批事项，再精简一批审批事项，加强事中事后监管。建设行政审批电子监察系统和电子政务公共云平台，打造管理规范、功能完备、办事公开、信息共享、运行高效的综合政务服务体系。健全重大决策社会稳定风险评估机制，将其作为前置程序、刚性门槛。重大决策出台前向市委、市人大报告，向市政协通报。加强政府规范性文件起草，健全行政复议案件审理机制，规范执法自由裁量权，做到严格规范公正文明执法。强化诚信意识，言必信、行必果，打造诚信政府。

推进务实政府建设。扎实开展党的群众路线教育实践活动。牢记空谈误事，实干兴市。做到知实情、讲实话、干实事、求实效，不怕吃苦、不图安逸，抓紧每一天、抓好每件事，推动各项工作落实到位。坚持立足实际，把握市情，实行非均衡发展战略，集中精力抓主要矛盾和矛盾的主要方面，不断提高政府工作的指向性。坚持解放思想、更新观念、开放拉动，在发展理念、发展产业、发展措施上向东部地区看齐，学思路、学方法、学创新。既立足自身，差异发展、错位发展，更借助外力，积极承接、一步对接，不断提高同台竞技的水平。强化问题意识，推行“一线工作法”，把重心放在解决问题的一线。建立健全问题收集、交办、督办机制，一级抓一级、一级对一级负责。实行“一事一议一决”，着力打造敢担当、有作为的政府。

推进廉洁政府建设。严格落实党风廉政建设责任制，健全惩治和预防腐败体系。坚持用制度管权管事管人，把权力关进制度的笼子。建立完善全市统一的公共资源交易市场，凡在目录范围内的各类公共资源交易活动都要进入市场，推行交易电子化，以完善的规则和严格的制度，约束和规范权力运行和政府行为，从源头上预防腐败。加强作风建设，坚决纠正损害群众利益的不正之风。加强行政监察和审计监督，完善大督查机制。科学合理制定目标，严格考核，严肃问责。深化政务公开，提高公开实效。认真遵守“八项”规定，坚决反对“四风”，厉行节约，反对浪费。牢固树立“过紧日子”和“会过日子”思想，严格控制一般性支出，坚决压缩“三公”经费，提高财政资金使用绩效，创新资金使用方式，强化预算约束，健全完善公开、透明、规范、完整的全口径预算制度，把有限的资金更多地用到保障改善民生、引领科技创新、做大经济体量等最迫切的事情上。

各位代表，马鞍山正处在转型升级、加快发展的关键时期，面临的任务艰巨而繁重，肩负的责任重大而光荣。让我们在省委、省政府和市委的坚强领导下，以强烈的责任感和使命感，自我加压、真抓实干、齐心协力、奋力拼搏，为早日建成更高水平小康社会、在全省率先实现现代化而努力奋斗！
